# The Role of Substrate Topography and Stiffness on MSC Cells Functions: Key Material Properties for Biomimetic Bone Tissue Engineering

**DOI:** 10.3390/biomimetics7010007

**Published:** 2021-12-31

**Authors:** Foteini K. Kozaniti, Despina D. Deligianni, Margarita D. Georgiou, Diana V. Portan

**Affiliations:** 1 Laboratory of Biomechanics & Biomedical Engineering, Department of Mechanical Engineering & Aeronautics, University of Patras, 26504 Patras, Greece; fkozaniti@upatras.gr (F.K.K.); deliyian@upatras.gr (D.D.D.); maggie.georgiou@yahoo.gr (M.D.G.); 2 Center for Advanced Medical and Pharmaceutical Research (CCAMF), University of Medicine, Pharmacy, Science and Technology of Targu Mures, 540000 Targu Mures, Romania

**Keywords:** polymeric biomaterials, substrate stiffness, mesenchymal stem cells, biocompatibility, bone tissue regeneration

## Abstract

The hypothesis of the present research is that by altering the substrate topography and/or stiffness to make it biomimetic, we can modulate cells behavior. Substrates with similar surface chemistry and varying stiffnesses and topographies were prepared. Bulk PCL and CNTs-reinforced PCL composites were manufactured by solvent casting method and electrospinning and further processed to obtain tunable moduli of elasticity in the range of few MPa. To ensure the same chemical profile for the substrates, a protein coating was added. Substrate topography and properties were investigated. Further on, the feedback of Wharton’s Jelly Umbilical Cord Mesenchymal Stem Cells to substrates characteristics was investigated. Solvent casting scaffolds displayed superior mechanical properties compared to the corresponding electrospun films. However, the biomimetic fibrous texture of the electrospun substrates induced improved feedback of the cells with respect to their viability and proliferation. Cells’ adhesion and differentiation was remarkably pronounced on solvent casting substrates compared to the electrospun substrates. Soft substates improved cells multiplication and migration, while stiff substrates induced differentiation into bone cells. Aspects related to the key factors and the ideal properties of substrates and microenvironments were clarified, aiming towards the deep understanding of the required optimum biomimetic features of biomaterials.

## 1. Introduction

The phenotypic expression and function of stem cells are regulated by their integrated response to variable microenvironmental cues [1]. The extracellular matrix (ECM) varies in composition, as well as in physical parameters, including stiffness and topography. With the development of tissue engineering and regenerative medicine, the potential effects of ECM physical properties on cell behavior at the cell–matrix interface are drawing much attention. The development of advanced biomaterials with desired characteristics that can adjust the biointerface between cells and the synthetic substrate, and mimic the natural properties of the ECM, becomes a great hotspot.

**Figure 1:**
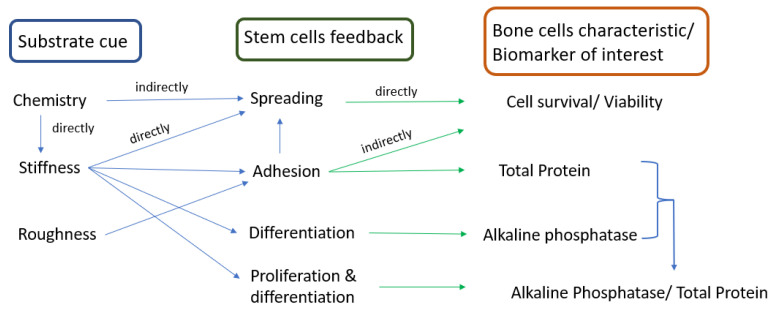
Correlation between substrate characteristics, stem cells feedback, and the corresponding biomarker.

Cellular responses are influenced by a multitude of factors in between which the substrate; some outstanding substrate characteristics which affect the quality of the biointerface and consequently cells faith are chemistry [2], texture/topography [3], scale [4], and stiffness [5]. Interplay or combination of such parameters is often considered, too [6]. Decoding the mechanisms and measuring the extent to which each of these parameters affects the cell population faith is an elaborate task that depends on the cell type and the interaction with each substrate parameter and requires analyses of complex microenvironmental lab systems. As indicated in the scheme in Figure 1, it has been shown that in the case of stem cells, chemistry has an indirect effect on cell spreading as it is directly influencing substrate stiffness [7] which in turn affects cells spreading; appropriate cells spreading results in a high cell survival rate and cell viability. Analyzing the circuit, we may find a direct or indirect way to correlate the substrate cue with stem cells feedback and the biological marker which characterizes the cell response. Thus, stiffness directly affects cell adhesion which obviously has a pronounced effect on cell spreading. Cell adhesion should be intermediary in order to allow adherence and adaptation to the substrate, and, at the same time, to allow sufficient cell freedom for division and communication with other cells; adhesion, therefore, indirectly affects cell survival and viability. Differentiation and proliferation are also influenced by stiffness, and an increased ratio of these two parameters indicates an appropriate metabolism of cell population. While stiffness induces differentiation [8], an appropriate roughness induces adhesion. In the case of metals which, due to their nature, enable the release of adhesion proteins in the ECM, roughness is not as important as it is for polymeric substrates, where there is a need of surface processing to promote adhesion. 

Topography is a key regulator of cell response. Cells in living organisms are surrounded by, and in contact with, nanoscale topographic interfaces, commonly known as basement membranes, which are composed of a complex mixture of structures and shapes, with sizes on the order of nanometers [9]. Studies on nanostructures have proved that they can modulate cellular responses, including cell morphology, motility, proliferation, protein abundance, adhesion, and gene regulation [10,11,12,13]. The scaling parameter must also be considered when planning to design biomimetic biomaterials. Furthermore, stem cell fate has been also linked to the mechanical properties of the underlying substrate, ultimately leading to downstream biological response. To evaluate the importance of substrate elasticity in biomaterial design, it is critical to test a wide variety of substrata that span physiologically relevant ranges of elasticity. The critical Young’s modulus of scaffolds for bone tissue engineering is in the order of ΜPa; despite that, many studies focus on low elasticity matrices, in the order of kPa, due to manufacturing issues that are encountered when fabricating higher-elasticity moduli substrates [9,14,15,16,17]. Few studies have focused on investigating the effect of higher stiffnesses substrates on cells. For instance, Olivares-Navarrete et al. found that human osteoblasts expression levels of osteoblastic genes increased only on the stiffer surfaces (310 MPa) [18]. Higher matrix moduli in 2D and 3D structures have generally been found to promote osteogenesis [11]. Tissue scaffolds are computationally designed and fabricated to match native bone properties. Studies confirmed that bone remodeling is at its best when the scaffold elastic tensor matches or is slightly higher than the elastic properties of the host bone [19]. However, to manufacture the numerically designed scaffolds, innovative technologies and new biocompatible materials are needed to accelerate the development of available grafting techniques. The traditional orthopedic biomaterial is titanium (Ti), which is biocompatible and strong, but this metal is much stiffer than bone (leading to stress shielding) and does not resorb [20]. While the cortical tissue has an elasticity modulus between 17 and 20 MPa, Ti has an elasticity modulus around 110 GPa. Polymers, on the other hand, have poor structural integrity and efforts are constantly made to improve their mechanical properties, such as, for instance, their elasticity moduli, above few kPa. Classic manufacturing technologies (e.g., injection molding) do not allow the fabrication of structural biomedical polymers with biomimetic features. Novel technologies offer new perspectives for the adjustment of the overall properties of biomedical polymers. Studies started years ago and are focused on the route for the development of polymers with a very broad range of properties similar to those of natural tissues [21]. Moreover, composites with a polymeric matrix are widely investigated [22,23] due to the many advantages they may introduce: (i) enhanced bioactivity, (ii) adequate control of the scaffold degradation rate, and (iii) enhanced mechanical properties and structural integrity of scaffolds. The approach is always multidisciplinary, involving manufacturing and processing technologies of biomedical polymers, characterization of their key properties, and biological in vitro evaluation with cell cultures.

As the potential range of stem cell applications in tissue engineering continues to grow, appropriate scaffolding choice is necessary to create tightly defined artificial microenvironments for each targeted organ. These microenvironments determine stem cell fate via control over differentiation [24]. The novel approach presented in this manuscript consists of the combination of advanced fabrication techniques, material processing, and characterization of substrates, followed by the correlation and the interpretation of the outcome in relation to specific cell markers, to determine the key factors in the elaboration of optimum biomimetic bone scaffolds. While the majority of studies consider the mechanical evaluation and the biological assessment of biomaterials as different sections, within the present research, a complete cycle is presented to finally conclude which manufacturing technique is more suitable for the targeted material to be used as a bone graft. Moreover, a wide range of elasticity moduli of the produced substrates has been achieved by using the same materials and coatings, obtained with two different fabrication techniques. This highlights the impact of the manufacturing method on the overall characteristics of the biomaterial. 

Within the present work, different PLA and CNTs-reinforced PLA substrates were fabricated with two technologies, solvent casting and electrospinning, respectively, to observe the influence of their mechanical properties and topographies on MSCs behavior. The role of substrate key characteristics on stem cells response was the main concern. Scaffolds’ morphologies and mechanical properties were investigated, followed by evaluation of their potential to induce proliferation and differentiation in embryonic mesenchymal progenitor cells. The goal was to select the appropriate topography–stiffness combination for scaffolds that promotes bone tissue formation. 

## 2. Materials and Methods

### 2.1. Materials and Fabrication Methods

Polycaprolactone (PCL) pellets with a molecular weight of 80,000 g/mol, glacial acetic acid of 99.8% purity, and acetone were purchased from Sigma-Aldrich (St. Louis, MO, USA). Pristine MWCNTs of purity ≥ 98.5%, external diameter 20–40 nm, and length ≥ 10 μm suitable for bioapplications were supplied by Nanothinx S.A. (Patras, Achaia, Greece). The 1 mm thick titanium alloy specimens (Ti-6Al-4V), purchased from Plus Endoprosthetic AG, CH 6343, Rotkreuzhad, had the following composition: Al: 5.5–6.5%, V: 3.5–4.5%, Fe: max 0.25%, O: max 0.13%, C: max 0.08%, N: max 0.05%, H: max 0.012%, Ti: balanced. All other chemicals were of reagent grade.

The techniques of solvent casting and electrospinning were applied to manufacture the scaffolds. Four different substrates were fabricated: (1) PCL by solvent casting (PCL-SC), (2) CNTs-reinforced PCL by solvent casting (PCL-CNTs-SC), (3) PCL by electrospinning (PCL-ES), and (4) CNTs-reinforced PCL by electrospinning (PCL-CNTs-ES), as shown in Table 1. 

In the case of the *solvent cast* method, PCL-SC films were fabricated by mixing a 10% PCL solution in acetone and by gently heating while mixing in a stirrer for 3 h. The polymeric solution was casted in a glass mold. The PCL films were formed after the evaporation of the solvent. For PCL-CNTs-SC films, 1 wt.% carbon nanotubes (CNTs) were mixed in a stirrer in PCL matrix. Further on, to prepare the PCL-ES scaffolds, a 20% *w*/*v* solution of PCL was mixed in glacial acetic acid in a roller overnight. Heating at 40 °C was applied to increase the polymer dissolution. 

In the case of nanocomposite scaffolds made of CNTs-reinforced PCL (PCL-CNTs-SC or PCL-CNTs-ES), 0.5 wt.%. MWCNTs powder was added in the PCL solution. PCL-CNTs solutions were maintained in an ultrasound bath for 4 h to assure the appropriate dispersion of CNTs. All films were prepared within a maximum of 24 h after mixing the components of the solutions, to avoid the hydrolysis of the polymer by the acetic acid. To fabricate solvent cast scaffolds, 2 mL of each solution was poured into a 76 mm × 22 mm custom glass mold. The mold was left on a flat surface under a fume hood for 48 h to allow the solvent to evaporate. 

The *electrospinning* parameters were voltage—20 kV, distance between the needle tip and the collector—20 cm, and the flow rate—1 mL/h. Experiments were performed in ambient conditions. Temperature and humidity were both measured during each individual experiment and were in the range of 20–25 °C and 40–50% relative humidity (RH), respectively. 

Samples were prepared for characterization as follows: scaffolds were cut, maintained in PBS for 24 h, and sterilized in 70% ethanol solution for 2 h; finally, they were exposed to UV for 30 min. To maintain the same chemical profile, the substrates were coated with fibronectin protein. Fibronectin was purchased from Applichem (A8390), and a solution of 0.01 *v*/*v* was prepared. The specimens were coated with fibronectin solution for 1 h before cell seeding.

### 2.2. Surface Analysis 

The topography of the scaffolds was investigated using a scanning electron microscope (SEM) (JEOL-JSM 6300, Tokyo, Japan). For SEM analysis, samples were cut into a round shape (1 cm diameter), coated with gold, and examined with an accelerating voltage of 20 kV. Fiber diameter and surface pore size of the electrospun substrates were calculated using ImageJ software (National Institutes of Health, Bethesda, MD, USA). Static water contact angle measurements were taken using a CAM101 goniometer (KSV Instruments Ltd., Helsinki, Finland) according to ASTM Standard D5946-17 and D7334-08. For each scaffold, three measurements were performed for three samples. Tests were carried out at room temperature using an 8 μL drop of distilled water. Scaffolds were also tested after being immersed in culture medium for 24 h and dried for 24 h. 

An atomic force microscopy (AFM) device (Nanoscope IIIa, Veeco, CA, USA) was employed to determine the roughness and the 3D profile of the substrates. The surface roughness parameter round mean square (RMS) was calculated in tapping mode on 5 µm × 5 µm areas.

### 2.3. Mechanical Testing

Tensile tests were performed up to failure using a MiniMat 2000 Machine from Rheometric Scientific (Piscataway, NJ, USA). The mean Young’s modulus of elasticity and the ultimate stress at failure of the substrates were determined according to ASTM D 882-12. Testing samples had the dimensions 7 × 35 × 5 mm. To prevent slippage, sandpaper was attached to the grips on both sides. The free length was 12 mm, and the strain rate was 5 mm/min. All tests were performed at room temperature (N = 15).

### 2.4. Biological Tests

Human Wharton Jelly Mesenchymal Stem Cells (hWJ-MSCs) derived from umbilical cords were donated from the Biomedical Research Foundation (Academy of Athens). The isolation protocol has been previously described by Chatzistamatiou et al. [25]. Cells were incubated in appropriate culture medium to promote their osteogenic differentiation, consisting of MEM supplemented with 10% FBS, 2 mM l-glutamine, 1% *v*/*v* amphotericin B, 0.5% *v*/*v* gentamicin, 50 μg/mL l-ascorbic acid, 10 mM β-glycerophosphate, and 10^−7^ M dexamethasone (Sigma Aldrich). Substrates were cut into disks with 14 mm dimeter and placed in 24-well plates. Cells were seeded on the testing substrates at a density of 50,000 cells/cm^2^. Tissue culture plastic (TCP) was used as control material. The medium was regularly changed after post-plating, every 3 days. 

The *MTT reduction assay* was used to evaluate cells viability for different incubation periods. A 5 mg/mL solution of MTT (Sigma, Saint Louis, MO, USA) was diluted to a 1:10 ratio in serum-free medium, as indicated in the guidelines. Scaffolds with seeded cells were transferred to a new well, washed with serum-free medium, and incubated with 500 μL MTT solution for 3 h. Further on, the MTT solution was removed and 500 μL of DMSO was used to dissolve the formazan crystals. From each sample, 100 μL solution was added in a 96-well plate in triplicate, and the absorbance was read at 570 nm. *Cell differentiation* was evaluated by alkaline phosphatase (Sigma Aldrich S0942) levels. *Alkaline Phosphatase* (ALP) activity was measured using a colorimetric assay after 3 and 7 days of cell incubation on substrates. *Total protein* was measured using a Total Protein Detection Kit (Cayman, Ann Arbor, MI, USA) according to the manufacturer’s instructions. ALP per total protein ratio was used to estimate the differentiation per total protein content of each sample. *Mineralization* was evaluated by Alizarin Red S staining after 14 and 21 days of culture. Cells were fixed with 4% paraformaldehyde for 15 min. For staining, scaffolds were immersed in a 2% alizarin red S (Sigma) solution for 20 min and rinsed with distilled water until no more dye was released. Sample micrographs were obtained using a stereoscopic microscope. Results were also quantified according to the procedure described by Gregory et al. [26]. Briefly, each scaffold was incubated with 10% acetic acid for 30 min and the supernatant was transferred to an Eppendorf tube which was then vortexed and heated at 85 °C for 10 min, while being covered with parafilm to avoid evaporation. After complete cooling, the pH was adjusted using 10% ammonium hydroxide and the absorbance was read at 405 nm. Immunofluorescent staining method was used to visualize the spreading of cells on the substrates after 7 and 14 days of culture. The specimens were removed from culture medium, rinsed twice with PBS, fixed with 4% paraformaldehyde, permeabilized with 0.2% Triton-X, and blocked with 5% bovine albumin. The Actin Cytoskeleton and Focal Adhesion Staining Kit (FAK100; Millipore, Darmstadt, Germany) were used to visualize the cells’ actin filaments and nuclei, respectively. Images were taken using fluorescence microscopy.

All results are expressed as mean ± standard deviation. Statistical analysis was performed using Student’s *t*-test and differences were considered significant when *p* < 0.05.

## 3. Results

The present investigation was performed in such a way to cover a wide range of characteristics of the substrate, of the cell population and the system involving both substrate and cells, as well as to further determine their type of interaction. The surface characteristics of a substrate are crucial for the wellbeing of the cell population. This is because within the first hours until days, cells are only influenced by the surface features of the substrate. Properties such as texture, scaling, roughness, and surface energy decide for the development, morphology, adaptation of cells and consequently, for the biointegration in the host body. As soon as a biomedical implant is placed in the human body, a cascade of biological reactions immediately takes place on the implant surface and continue to occur in a dynamic state for the entire lifetime of the implant [27]. As shown in the sections below, surface energy and mechanical properties also tremendously influence the development and functions of stem cells, as well as their differentiation. Concerning cell population indicators, a test was conducted to measure their viability as division, multiplication, and appropriate migration are the very important functions that will lead to new tissue formation. Alkaline phosphatase, total protein, and their ratio were investigated to understand the balance between proliferation, adhesion, and differentiation, and therefore to appreciate the extent to which the manufactured substrates may be used for stem cells differentiation into bone cells. Mineralization was studied too, because the bone mineralization process is essential for the hardness and strength of bone. If this process is not properly regulated, the resulting mineralization will be either insufficient or excessive. As a consequence, the quality of bone tissue can be compromised [28]. Finally, cells staining was performed to observe their spreading on the different substrates. 

### 3.1. Surface Analysis and Roughness

**Figure 2:**
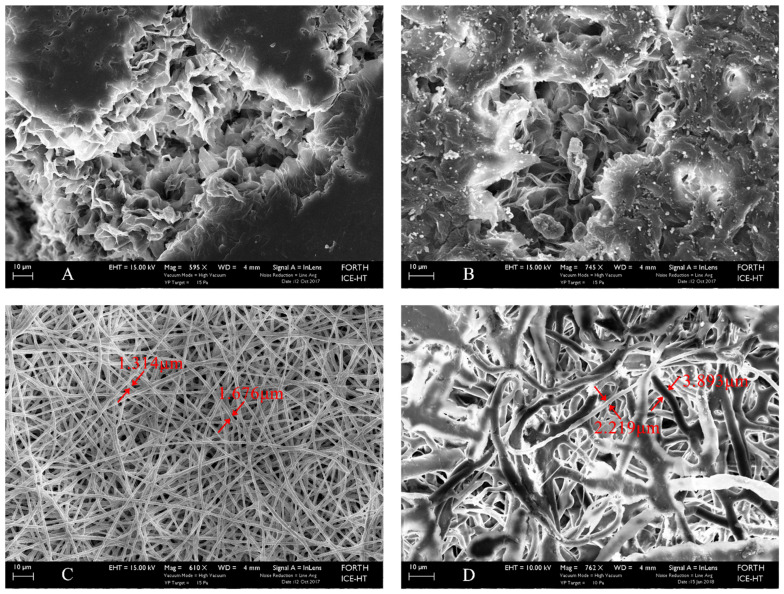
SEM images of scaffolds: (**A**) PCL solvent cast film (Mag 595×); (**B**) CNTs-reinforced PCL solvent cast film (Mag 745×); (**C**) PCL electrospun film (Mag 610×); and (**D**) CNTs-reinforced PCL electrospun film (Mag 762×).

Comparing Figure 2A,B with Figure 2C,D, one may observe the difference in microstructure between the films synthesized with the solvent casting method vs. those manufactured by electrospinning. While solvent casting films present an undefined structure, with several discontinuities and nonuniform distribution of pores, the electrospun films are formed by entangled fibers, uniformly distributed, creating a visibly homogeneous microstructure. Generally, porous surfaces positively influence cells feedback. It was found that microgrooved substrates of an implant surface did not promote cells proliferation or osteogenic differentiation of hMSCs into osteoblasts, but they did favor the collective cells migration and thus subsequent healing of wounds. In addition, it has been stated that to engineer functional tissue equivalents that closely mimic the unique properties of native tissues, it is necessary to develop strategies for reproducing the complex, highly organized structure of these tissues [29]. As it is shown and discussed in the sections below, both solvent cast and electrospun films promote cells growth and improved cellular response compared to titanium substrates, which is by now the traditional orthopedic metal. However, due to their tissue-like features, electrospun substrates enhance cells feedback in terms of development (morphology), division, and multiplication.

Fiber diameter, surface pore size, and internal pore size are listed in Table 2. Measurements for the solvent casting scaffolds were performed only at their surface. Their internal structure is not homogeneous, pores are not regular or uniformly distributed, and cannot be measured. Compared to the electrospun fibers, surface pores of the solvent casting scaffolds are much reduced in dimension; the addition of CNTs leads to formation of pores three times longer in diameter. In the case of electrospun scaffolds, the bulk electrospun ones compared to those reinforced with CNTs have also been designed with larger fibers to avoid obstruction due to the inclusion of the nanoreinforcement. Consequently, the entire microarchitecture of the reinforced films has been shifted to larger dimensions of both surface pore sizes and bulk pore sizes.

The values presented in Table 2 show that the mesh created by the fibers forms large enough pores to allow cells to migrate within the underneath layers. Stem cells in scaffolds may reach an average of 12 μm length [30]. Besides the cell itself, medium and nutrients may also pass between the layers and assure an appropriate environment along the thickness of the substrate. 

**Figure 3:**
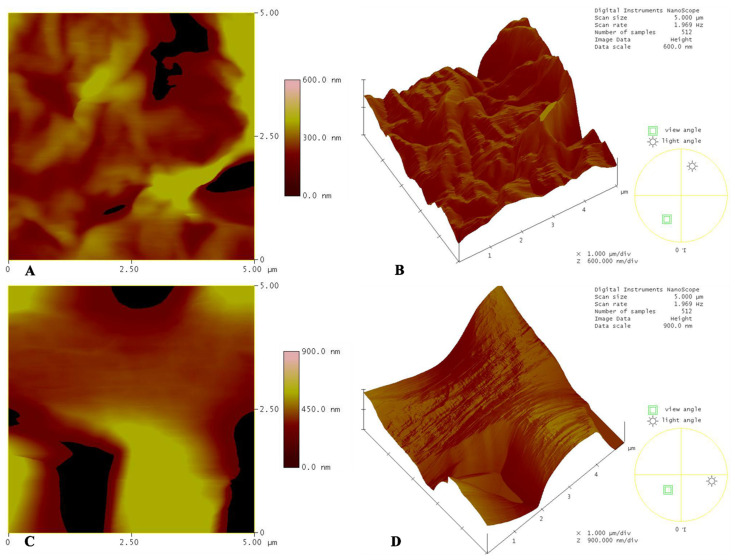
Topographical AFM analysis and 3D patterns of scaffolds: (**A**) Roughness of PCL solvent casting scaffold on 5 × 5 scan area; (**B**) 3D pattern of PCL solvent casting scaffold on 5 × 5 scan area; (**C**) Roughness of PCL electrospun scaffold on 5 × 5 scan area; (**D**) 3D pattern of PCL electrospun scaffold on 5 × 5 scan area.

Atomic force microscope (AFM) analysis was further performed, as it can provide quantitative information regarding the topography of a surface. Many functional properties, e.g., adhesion, hydrophobicity, and contact conductance of a coating or surface are directly related with its roughness [31]. Figure 3 shows the topographical AFM analyses of the PCL solvent casting and electrospun scaffolds. We may observe the average values of the measured heights in Table 3. It is evident from both measurements, and from the topographical profile, that the roughness of the electrospun scaffolds is much more pronounced (almost three times) than that of the solvent casting scaffolds. Considering that stem cells and bone cells are anchorage-dependent, and that cell adhesion, beginning from the formation of cell adhesion complexes, is a prime example of the response of cells to a given topography [32], it becomes clear that the increased contact area between cells with the substrate favor key cellular processes. Thus, the electrospun scaffolds provide a positively modified substrate for the cells with respect to roughness, compared to the solvent casting material, and, as a consequence, promote their adhesion. 

### 3.2. Contact Angle

Contact angle measurements were performed after maintaining for 10 s the drop of water on the scaffold surface. Two groups of samples were tested: (i) control scaffolds and (ii) scaffolds after sterilization and immersion in culture medium, for 24 h. For the second group of tested scaffolds, before measuring the contact angle, the scaffolds were sterilized and then immersed in culture medium for 24 h. Different times of measurement were taken into consideration (0, 30, 60 s), to observe the abrupt jump in contact angle values. The number of test specimens was at least nine for each case. The values of the contact angle for all the specimens and for different time durations are given in Table 4.

**Figure 4:**
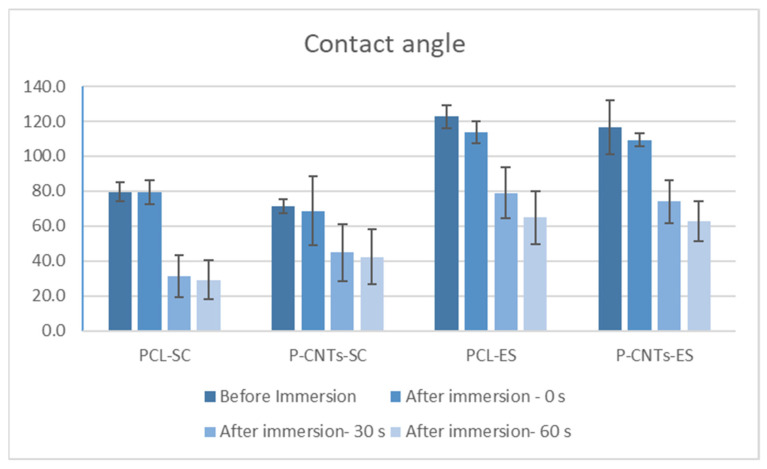
Contact angle in scaffolds before and after immersion in culture medium.

As observed in the diagram in Figure 4, immersion in culture medium showed that all the scaffolds are hydrophilic. In vivo, a thermoplastic hydrophilic substrate will absorb considerable body fluid amount within the first minutes after implantation. A recent study demonstrated that surface hydrophilicity cannot be correlated well with the osteoblast-like MG-63 cell adhesion and proliferation [33]. Others stated that hydrophilicity confers to biomaterials suitable characteristics to favor cellular adhesion and colonization [34]. However, a combination of different parameters will decide cells fate; texture, scaling level, and organization of the surfaces are also important. Conclusions may be drawn only after observing and comparing cells development on the different substrates. 

Compared to the solvent casting films, electrospun films (ES) are less hydrophilic, which is due to their fibrous structure, and the reduced contact area with the drop. No considerable differences of hydrophobicity/hydrophilicity exist between the bulk and the CNTs reinforced substrates, in both cases of solvent casting and electrospinning. This shows that the internal composite structure of the substrates does not affect its surface properties, which is an advantage and was the target of the present investigation. Therefore, elasticity modulus and other mechanical properties of the substrate can be tuned without important changes in its surface characteristics. This in turn allows to compare cells’ responses to the mechanical cues introduced by the substrates. As seen in the diagram in Figure 4, the contact angles of the substrates produced by solvent casting have lower values. An important consequence of the pronounced hydrophilicity may be the high biodegradation rate of the scaffold. Electrospun substrates may therefore be more resistant to degradation for longer immersion periods of time.

### 3.3. Mechanical Properties

Many excellent review articles discuss cellular responses to substrate stiffness [35,36,37,38] or topography [39,40]. However, despite similarities in phenotypic manifestations, the interwoven effects of stiffness and topographical cues on cell behavior and the mechanisms and molecular processes underlying stem cell differentiation and cell fate determination have not been completely described; case studies will considerably contribute to elucidating some of these issues. The challenge associated with the evaluation of polymeric biomaterials is related to the fabrication of substrates with the same chemical composition but different texture and mechanical properties, to assess the influence of each parameter on cell population behavior. This has been achieved within the present investigation by manipulating two different fabrication techniques (solvent casting and electrospinning) to produce substrates made of bulk PLA and CNTs-reinforced PLA, that were further coated with fibronectin.

**Figure 5:**
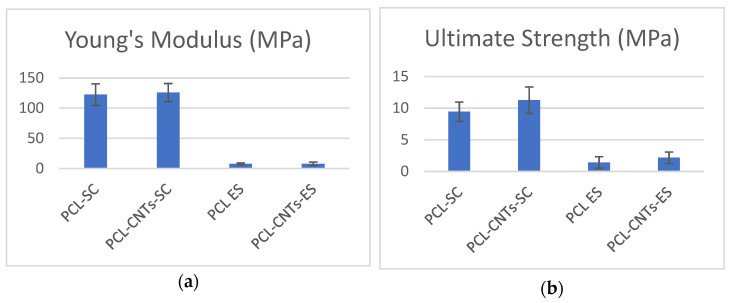
(**a**) Elasticity modulus of the films prepared by solvent casting method vs. films prepared by electrospinning and (**b**) maximum strength.

Comparing the values of the mechanical properties (Young’s modulus and ultimate strength) of solvent casting substrates and electrospun substrates (Table 5, Figure 5), we immediately observe the pronounced difference, showing how the manufacturing technique of a material dramatically influences its final mechanical properties. Solvent casting substrates present a Young’s modulus 15 times higher than that one of the electrospun substrates. This is because of the fibrous nature of the electrospun substrates, which breaks easily compared to the compact solvent casting scaffolds. On the other hand, it may be observed that the CNTs-reinforcement lightly increased the mechanical properties of the PCL, especially the maximum strength of the electrospun substrates. 

Analyzing the diagrams in Figure 5, we see the prominent differences between the mechanical properties of the casting solvent and the electrospun materials. Thus, we may deduce that the mechanical properties of the substrate are a decisive factor for any significant change in cells response on the various substrates. 

### 3.4. Cell Viability (MTT Assay)

**Figure 6:**
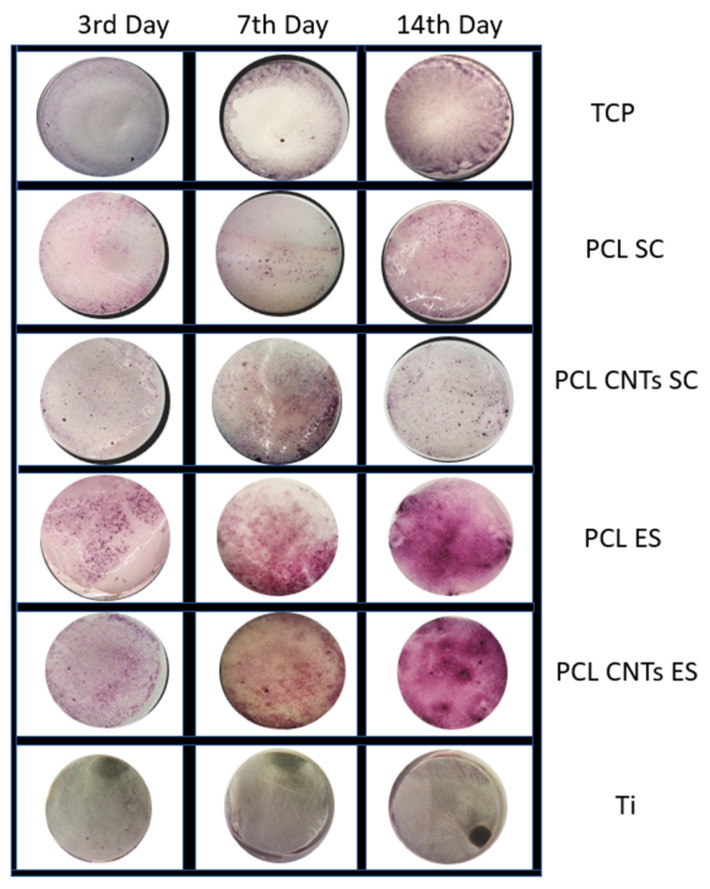
Cell proliferation: Quality MTT 3, 7, and 14 days of incubation on the different substrates.

MTT assay was used to evaluate cells viability. Substrates’ color and texture after performing the assay may be seen in Figure 6. No pronounced difference exists between the PCL and the CNTs-reinforced PCL substrates, independently of the fabrication technique. Reactions were much more evident in the case of the electrospinning films, especially after 14 days of culture. Apparently, this indicates an increase in cells viability on these types of substrates. However, the quantification of the MTT is compulsory for further analysis of the samples.

**Figure 7:**
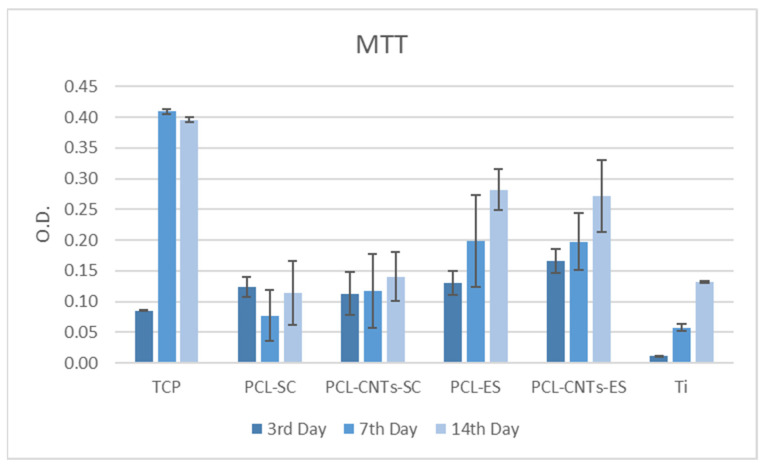
Quantification of MTT levels on all substrates after 3, 7, and 14 days of incubation.

After the third day of incubation, cells proliferated on electrospun CNTs-reinforced PCL more than on the other substrates (Figure 7). A significant difference may be seen when comparing the polymeric substrates with titanium. Titanium does not promote cells proliferation, as it may be seen. Further on, electrospun substrates are more efficient compared to the solvent casting substrates. When comparing all substrates, we observe that cells viability and proliferation are enhanced on the less-stiff materials, especially after 14 days of incubation. This is due to the biomimetic structure of these materials, which are closely imitating the natural microenvironment of the cells.

It is assumed that cells behavior becomes more stable after longer incubation periods. This is also related to MSCs differentiation into osteoblasts, over time, which is influenced by several factors, in between which the substrates characteristics fall, too. Stiffer substrates induce faster differentiation as they mimic the natural bone tissues. On the other hand, maximum proliferation level is expressed at earlier stages, in MSCs or preosteoblasts, and decreases after cells differentiation. It was found that cell proliferation and differentiation show a remarkable inverse relationship. Precursor cells continue division before acquiring a fully differentiated state, while terminal differentiation usually coincides with proliferation arrest and permanent exit from the division cycle. Mechanistic insight in the temporal coordination between cell cycle exit and differentiation has come from studies of cells in culture and genetic animal models [41]. In the present investigation we also observe that the stiffer films prepared by solvent casting induce differentiation and reduced proliferation, while the fibrous electrospun film determines a high rate of proliferation in MSCs, as further proved below.

### 3.5. Alkaline Phosphatase and Total Protein Levels

**Figure 8:**
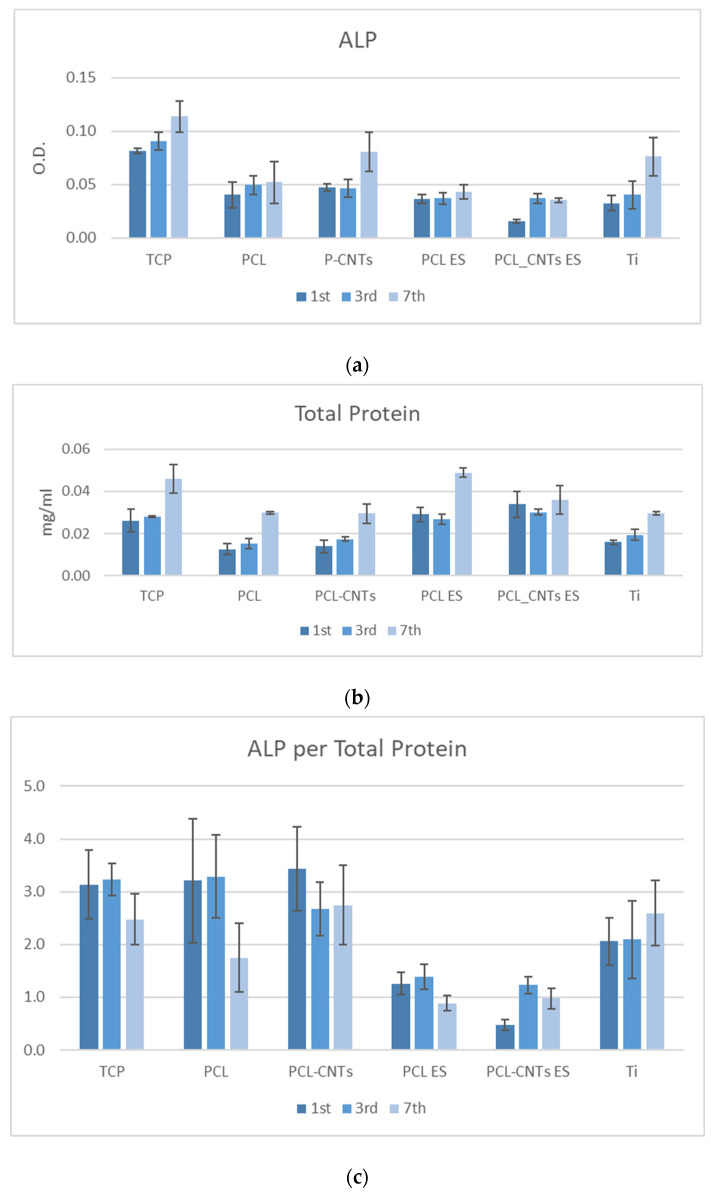
Levels of (**a**) alkaline phosphatase, (**b**) total protein, and (**c**) alkaline phosphatase per total protein in cells after 1, 3, and 7 incubation days.

Proliferation capacity of MSCs was quantified by alkaline phosphatase (ALP) activity. ALP is a key component of bone matrix vesicles because of its role in the formation of apatitic calcium phosphate, and it is an early indicator of immature osteoblast activity [42,43]. Although cells generate the enzyme in several tissues—liver, kidney, placenta, etc., elevated levels of ALP exist in bone tissue, typically several days prior to neomineralization and during the initial phase of bone matrix deposition. In the present study, the alkaline phosphatase test indicated an increased proliferation in cells on all the substrates, with time, which is a good indicator of cell metabolism (Figure 8a ALP). 

Proteins are known markers of osteoblasts phenotype [44,45] and are closely linked to cells adherence to a substrate [46]. Total protein levels were measured as an indicator of cell differentiation after 1, 3, and 7 days of incubation. The total protein levels in cells on electrospun substrates may be seen in Figure 8b. Total protein levels on electrospun PCL and CNTs-reinforced PCL were remarkably increased compared to those on the solvent cast scaffolds (Figure 8c), which is related to the mechanical properties of the substrate, and to its topography, to be further discussed in relation to the overall results of the study. It should be noted that after 7 days of incubation, the values of total protein in cells on TCP, CNTs-reinforced TCP, and titanium are almost similar. 

The ALP/total protein level was calculated, being an indicator of the balance between the two processes—proliferation and differentiation—in cells (Figure 8c). After 7 days of incubation, the TCP, solving casting CNTs-reinforced PCL, and titanium generated an almost similar ALP/TP ratio in cells, showing that there is an adaptation to the substrates which depends on its higher stiffness, which is the only common feature of these three substrates.

### 3.6. Mineralization (Alizarin Red Assay)

**Figure 9:**
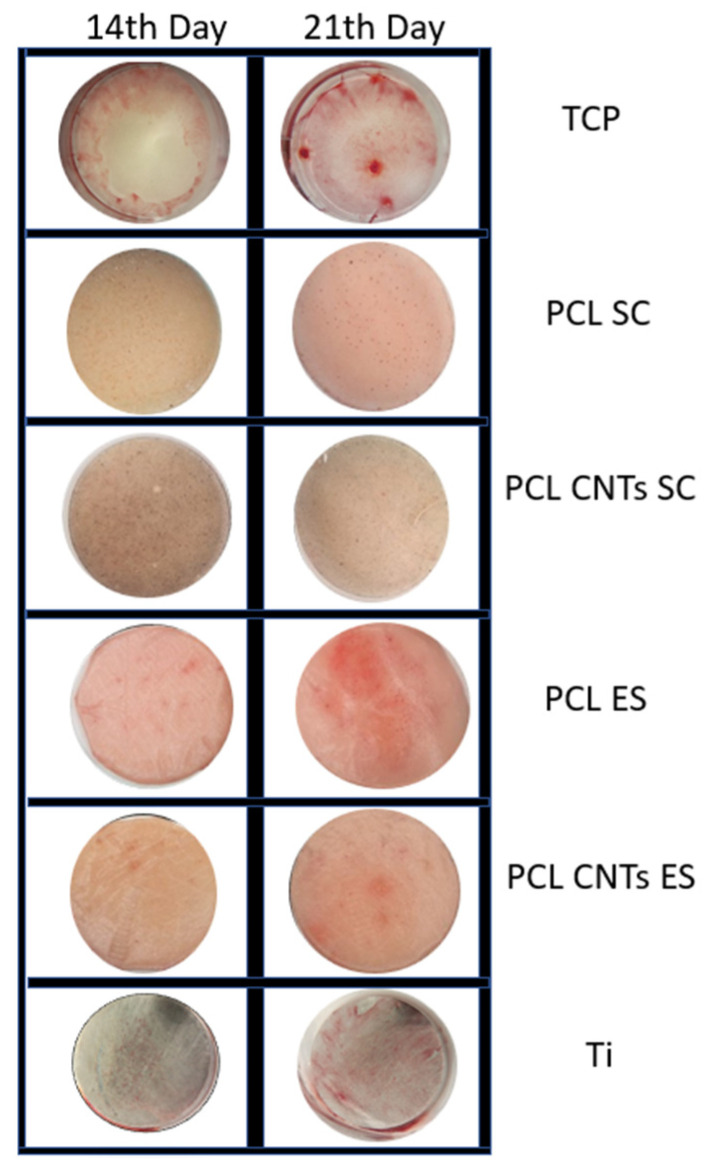
Quality alizarin red test on 14th day and 21st day for substrates with both high and low elasticity modulus.

Mineralization patterns were examined by alizarin red staining. On all scaffolds, the mineralization was remarkably enhanced compared to the TCP control substrate. Mineralization levels were low, but similar to those that Klontzas et al. [47] reported for umbilical cord blood mesenchymal stem cells. In Figure 9, alizarin staining may be observed for different substrates for long incubation periods (14 and 21 days). 

**Figure 10:**
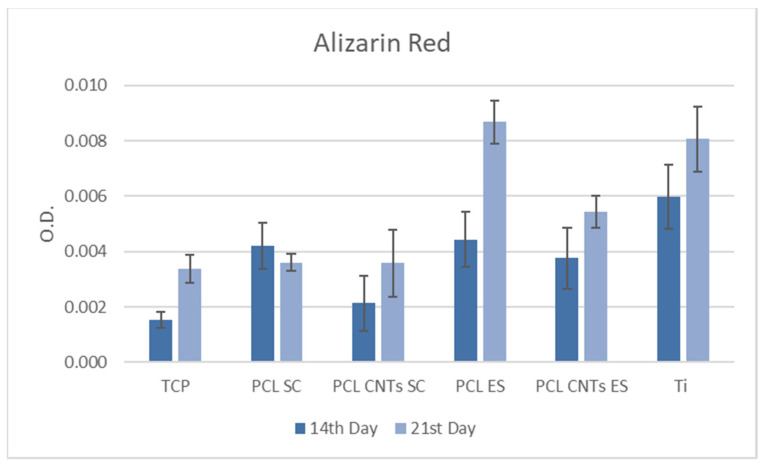
Quantity alizarin red test at 14 and 21 days of incubation on substrates.

The quantified alizarin red values may be seen in the diagram in Figure 10. Mineralization is considerably higher in cells on electrospun scaffolds compared to the other substrates, which agrees with the study of Ruckh et al. [43]. 

### 3.7. Cell Staining

**Figure 11:**
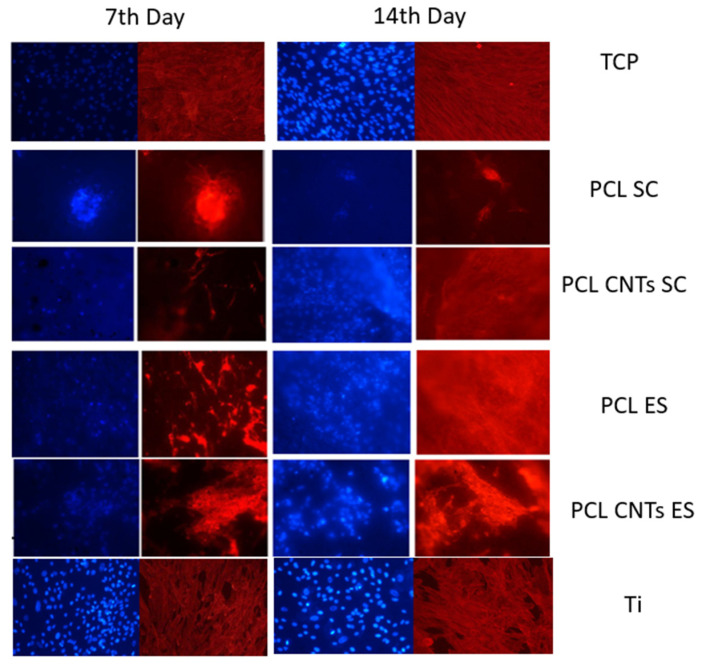
The 7th and the14th day of culture: Cells’ nucleus (stained with DAPI (blue)) and Cytoskeleton Actin (stained with phalloidin (red)) on PCL solvent cast, PCL-CNTs solvent cast, PCL electrospun, P-CNTs electrospun, and titanium, respectively (magnification 10×).

MSCs’ spreading was investigated by staining the nucleus and the cytoskeleton after 7 and 14 days of incubation. In Figure 11, representative images of cells on the different substrates may be observed. On titanium, as well as on TCP, cells spread uniformly, while they tend to agglomerate on all the other substrates. The two types of substrates, flat vs. scaffold, assure a different environment for the cells. On the flat and smooth substrates, cells stay on the surface, and they are homogeneously distributed in all directions, covering the entire area of the sample. On the opposite, a scaffold confers a complex 3D structure and, due to its entanglements, determines the formation of cells agglomerates. 

Wang’s group observed reduced cell spreading on relatively stiffer substrates [48]. However, the substrates in the present study differ not only with respect to their modulus, but also their overall structure. In this case, it cannot be stated that the reduced spreading on the polymeric scaffolds compared to the metallic one is because of their mechanical properties, but rather because of their architecture. On the other hand, analyzing cells on the polymeric scaffolds, we may see pronounced fluorescence and increased cell number on the reinforced PCL compared to the bulk material. According to Fu et al. [49], on rigid microposts, hMSCs were well spread with prominent, highly organized actin stress fibers and large focal adhesions. In contrast, cells on soft microposts had a rounded morphology with prominent microvilli, disorganized actin filaments, and small adhesion complexes. Overall, these observations suggested that cell shape, focal adhesion structures, and cytoskeletal tension were tightly coupled systems involved in rigidity sensing.

### 3.8. Comparation of Cumulated Results

Table 6 shows results of MSCs feedback to the four types of substrates, in terms of viability, proliferation, differentiation, and mineralization. Previously, it has been stated that a substrate having a 30 MPa elasticity modulus can induce osteogenic differentiation, while an elasticity modulus of 7.1 MPa determines chondrogenic differentiation [24]. Comparing the values in Table 6, we observe that, indeed, the level of alkaline phosphatase/total protein in cells on the substrates with higher elasticity modulus in the range of 120 MPa is much higher than in cells on less stiff substrates. This value is increased also on titanium, and therefore results are in agreement with previous studies. 

The ALP is an indicator of stem cells differentiation into preosteoblasts while the total protein level is related to cells adhesion and interlocking with the substrates. Osteoblasts’ main function is to adhere to the substrate and build the tissue network. Higher ALP/TP levels indicate that cells regulate their function and adapt to the substrate. It has been stated that there is a strong osteogenic response of the umbilical cord-MSCs (UC-MSCs) to the more rigid substrate, confirmed by mineralization staining, which is attributed to the fact that such a substrate best mimics the natural bone microenvironment. Within the present investigation, no significant mineralization difference was observed in cells on the substrates with high vs. low elasticity modulus. This may be because, as affirmed by Olivares-Navarrete et al. [18], to achieve a stable osteoblast phenotype in MSCs grown on plastic, an extensive time in culture is required to develop multilayered nodules, as is the use of media supplements for as long as three weeks to support mineral formation within the nodules. Extended incubation time might lead to significant differences of the mineralization in cells depending on substrates properties. 

**Figure 12:**
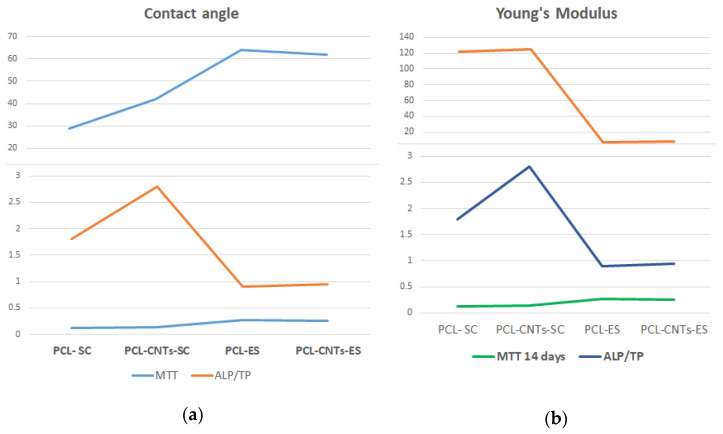
Values of (**a**) contact angle (blue line), MTT (green line), and ALP/TP (orange line) for the four manufactured substrates and titanium and (**b**) Young’s modulus (orange line) for the four manufactured substrates together with MTT (green line) and ALP/TP (blue line).

To better compare and interpret results, suggestive values of the different tests were selected and are plotted in the graphs in Figure 12. All fabricated substrates were hydrophilic. However, comparing substrates, we can separate them in two groups: solvent casting hydrophilic substrates and electrospun hydrophobic substrates. As seen in Figure 12, the MTT viability and proliferation test indicated a smooth increase in value in cells on hydrophobic (electrospun substrates). This was not the case for the ALP/TP level, which was much lower in cells on the electrospun substrates. However, as previously mentioned, Young’s modulus of the substrate is a key factor determining cells development. On the other hand, contact angle (surface energy) and elasticity modulus may produce a synergetic effect on cells population. As observed, electrospun substrates have high contact angle and low elasticity modulus. High contact angle means low surface energy. Surface energy is a fundamental material property that can influence cells’ behavior, especially when cells are incubated for prolonged periods of time. Previous reports proved that surface energy does not affect initial cell adhesion at 8 h. However, the rate of proliferation was linearly dependent on surface energy and increased with increasing hydrophobicity. Moreover, cells were significantly smaller on the most hydrophilic regions. Finally, results showed that fibronectin-mediated cell spreading and proliferation are dependent on surface energy [51]. The increased values of the MTT test on electrospun materials is therefore the result of the fibrous surface texture, the surface properties (surface energy), and the elasticity modulus. The viability and proliferation of cells is very much associated with the freedom of movement and communication between cells. The 3D structure of the electrospun scaffolds is also important. The low surface energy of the electrospun substrates allows cells to multiply and migrate easily. The 3D structure assures higher surface area for cell spreading and complex actin interconnection.

On the solvent casting substrates, the phenomenon is reverse. More precisely, these substrates are both hydrophilic and mechanically superior, with a much higher elasticity modulus. In addition, cells agglomerate on the substrate surface as there is no 3D internal structure. In this case, due to the limited space between cells, the interconnection between them and the adhesion to the substrates are stronger. As the formed network is biologically more robust, cells tend to differentiate faster; higher ALP levels are released for differentiation, and higher TP levels are specific to adhesion processes. 

To resume, as previously stated by others, softer substrates induce less differentiation of MSCs into bone cell progenitors. Some key surface and mechanical features of the substrate significantly influence cells’ feedback. These are the substrate structure (2D vs. 3D), the surface energy of the substrate, the texture of the substrate (roughness, fibrous), and the mechanical properties of the fabricated material. According to the performed measurements and the obtained results, the most influential factor is the mechanical one.

## 4. Conclusions

The aim of the present investigation was to determine the influence of substrate stiffness along with topography on cells’ development, proliferation, and differentiation, in order to design biomimetic scaffolds suitable for bone tissue engineering (BTE). Four substrates with various internal and external characteristics were fabricated from PCL and CNTs-reinforced PCL to assess MSCs response to the microenvironment. Two manufacturing techniques were used: a classic one (solvent casting) and a modern one (electrospinning). The CNTs are a good reinforcement for the PCL matrix, as an increase in the modulus of elasticity and in the ultimate strength was detected with their addition independently of the manufacturing method. The solvent casting method produced compact films with less-homogeneous surface texture, but with an elasticity modulus in the order of 120 MPa. The electrospinning method produced 3D structures with entangled fibers, biomimetic surface texture, and very low elasticity modulus (7 MPa). The investigation underlines the importance of the chosen manufacturing technique for the addition of desired biomimetic features to biomedical polymeric materials. Electrospun substrates were less hydrophilic and consequently had a lower surface energy. The four key factors influencing cells fate were as follows:

1. Substrate structure: 2D for solvent casting method and 3D for electrospun substrates.
2. Surface texture: rough and nonuniform solvent casting substrates and fibrous electrospun substrates.
3. Surface energy: high for solvent casting substrates that are more hydrophilic and lower for electrospun substrates.
4. Elasticity modulus: very high for solvent casting substrates and very low for electrospun substrates.

The main cellular processes that determine new tissue formation and their correlation with the investigated substrates are as follows:

(a) Cells migration was enhanced on the 3D electrospun scaffolds due to higher area provided to cells for development and due to the low surface energy of the material.
(b) Cells proliferation is migration-dependent and was higher on the electrospun materials.
(c) Cells adhesion was improved on the solvent casting substrates due to their high surface energy and the high elasticity modulus.
(d) Cells differentiation from MSCs to osteoblasts is adhesion-dependent and was enhanced on the solvent casting substrates because of their mechanical properties (high elasticity modulus and strength) closer to the natural bone tissue.

Observing the above, we may establish different scenarios for the continuation of the present research. The electrospinning manufacturing technique is more suitable for synthetic vessels fabrication due to its less-hydrophilic nature and malleability; also, as it induces cells migration and proliferation, it will also be an appropriate candidate for synthetic skin manufacturing. This technique does not permit the fabrication of appropriate bone-replacing scaffolds as the resulted biomaterials have extremely low mechanical performance. The solvent casting fabrication method may be used for the production of stiff and porous scaffolds which induce differentiation of stem cells into bone cells. The ideal would be, however, to design and elaborate multilayered biomaterials with gradient properties along their stiffness. The electrospun substrates would be an appropriate coating which initially promotes cells recognition, multiplication, and proliferation. The degradation rate of the electrospun coating should be adjusted to self-removal (degradation) after no more than two to three days, until the foreign body response of the immune system is inactive. In the second phase of implant biointegration, the new cells should come in contact with the underneath substrate produced by solvent casting, which, due to its stiffness, will promote further cells’ proliferation and differentiation. 

The final conclusion is that biomimetic features of novel biomaterials should be programmed depending on the specific application. 3D electrospun materials could be successfully used in skin regeneration due to their similar characteristics with this natural tissue. For the differentiation of MSCs into bone cells, stiffer substrates with high surface energy are required. 

## Figures and Tables

**Table 1:** Fabricated scaffolds, employed materials, and abbreviations.

Method of Fabrication	Matrix	Reinforcement	Abbreviation
Solvent Casting	PCL	-	PCL-SC
Solvent Casting	PCL	CNTs	PCL-CNTs-SC
Electrospinning	PCL	-	PCL-ES
Electrospinning	PCL	CNTs	PCL-CNTs-ES

**Table 2:** Fiber diameter and surface pore size for electrospun and solvent cast films.

Parameter	PCL-ES	P-CNTS-ES	PCL-SC	PCL-CNTs-SC
Fiber Diameter (μm)	1.33 ± 0.45	3.13 ± 1.49	-	-
Surface Pore size (μm)	13.93 ± 3.16	29.47 ± 7.55	6.34 ± 3.26	18.13 ± 5.95
Internal Pore size (μm)	28.4 ± 24.79	24.57	-	-

**Table 3:** Roughness of PCL-SC and PCL-ES scaffolds on 5 µm × 5 µm and 20 µm × 20 µm scanning area.

Material	PCL-SC		PCL-ES	
Scan Area	Average	St. Dev.	Average	St. Dev.
μm^2^	nm		nm	
5 × 5	251.43	10.76	668.09	387.42

**Table 4:** Contact angle before and after immersion of the scaffolds in culture medium, for 24 h.

			Solvent Cast Method		Electrospinning	
	Time (s)		PCL-SC	P-CNTs-SC	PCL-ES	P-CNTs-ES
Before Immersion	10	Avg.	79.62	71.26	122.65	116.61
		St. Dev.	5.41	3.94	6.65	15.61
After Immersion	0	Avg.	79.31	68.6	113.59	109.37
		St. Dev.	7.07	19.73	6.37	3.84
	30	Avg.	31.22	45.01	79.07	74.07
		St. Dev.	12.03	16.33	14.79	12.42
	60	Avg.	29.16	42.38	64.93	62.76
		St. Dev.	11.12	15.61	15.09	11.36

**Table 5:** Mechanical properties of substrates (MPa).

		PCL-SC	PCL-CNTs-SC	PCL-ES	PCL-CNTs-ES
Young’s Modulus	Average	122.28	125.63	7.34	7.60
	St. Dev.	17.87	15.09	1.67	3.08
Maximum Strength	Average	9.43	11.27	1.41	2.17
	St. Dev.	1.53	2.10	0.92	0.91

**Table 6:** Cumulated results for the four tested substrates vs. titanium.

Cumulated Results	Substrate				
	PCL-SC	PCL-CNTs-SC	PCL-ES	PCL-CNTs-ES	Ti
Texture	compact	compact	fibrous	fibrous	compact
Contact Angle	29	42	64	62	64 [50]
Young’s Modulus	122 MPa	125 MPa	7.34 MPa	7.60 MPa	110 GPa
MTT 14 Days	0.12	0.14	0.27	0.26	0.13
ALP/TP 7 Days	1.8	2.8	0.9	0.95	2.6
Alizarin Red 14 Days	0.004	0.002	0.0041	0.0039	0.006

## Data Availability

Generated and analyzed data of this study are included in this published article and may be provided upon request from the authors.
